# Dietary Exposure and Risk Assessment of Multi-Mycotoxins (AFB1, AFM1, OTA, OTB, DON, T-2 and HT-2) in the Lebanese Food Basket Consumed by Adults: Findings from the Updated Lebanese National Consumption Survey through a Total Diet Study Approach

**DOI:** 10.3390/toxins16030158

**Published:** 2024-03-19

**Authors:** Maha Hoteit, Zahraa Abbass, Rouaa Daou, Nikolaos Tzenios, Lamis Chmeis, Joyce Haddad, Mohamad Chahine, Elham Al Manasfi, Abdulrahman Chahine, Omasyarifa Binti Jamal Poh, André El Khoury

**Affiliations:** 1 Food Sciences Unit, National Council for Scientific Research-Lebanon (CNRS-L), Beirut P.O. Box 11-8281, Lebanon; zahraaabbass33@gmail.com (Z.A.);; 2 Faculty of Public Health, Section 1, Lebanese University, Beirut P.O. Box 6573, Lebanon; 3 Centre d’Analyses et de Recherche (CAR), Unité de Recherche Technologies et Valorisation Agro-Alimentaire (UR-TVA), Faculty of Sciences, Campus of Sciences and Technologies, Saint-Joseph University of Beirut, Mar Roukos P.O. Box 17-5208, Lebanon; 4 Faculty of Nursing and Health Sciences, Notre Dame University-Louaize, Zouk Mosbeh, Zouk Mikael P.O. Box 72, Lebanon; 5 Faculty of Public Health, Charisma University, London EC1V 7QE, UK; nicolas@trccolleges.com; 6 Directorate of Preventive Healthcare, Ministry of Public Health, Beirut 6573, Lebanon; ingjoycehaddad@yahoo.com; 7 Biological and Chemical Technology, Kursk State Medical University, 305000 Kursk, Russia; 8 Arab Group for Scientific Research, Beirut 1103, Lebanon; 9 Obstetrics and Gynecology ICE, Kursk State Medical University, 305000 Kursk, Russia

**Keywords:** AFB1, AFM1, OTA, DON, contamination, exposure, risk assessment, FFQ, Lebanon

## Abstract

Mycotoxins have been linked to adverse health impacts, including liver cancer and kidney diseases. The objectives of the current study were to evaluate the dietary exposure of Lebanese adults to multi-mycotoxins (aflatoxin B1 (AFB1), aflatoxin M1 (AFM1), ochratoxin A (OTA), ochratoxin B (OTB), deoxynivalenol (DON), T-2 and HT-2) and to assess their associated health risks. Hence, a nationally representative sample of 449 participants aged 18-64 years old were interviewed to obtain their socio-demographic characteristics, food consumption data and exposure estimates. A food frequency questionnaire and 24 h-recall were used to collect data. The concentration of mycotoxins in all foods consumed by the participants was collected from previous national published studies. The estimated daily intake (EDI), the hazard quotient (HQ) and the margin of exposure (MOE) were calculated. The total exposure to AFB1, AFM1, OTA and DON was 1.26, 0.39, 4.10 and 411.18 ng/kg bw/day, respectively. The MOE to AFB1, AFM1, OTA and DON in the Lebanese food basket was 316, 1454, 3539 and 510, respectively, indicating high health-related risks. Per food items, the MOE to AFB1 was below 10,000 in cereals (466.5), mainly in rice (827.9) and Burgul (4868.5). Similarly, the MOE to OTA in cereals was 1439, in which bread (4022), rice (7589) and bulgur (7628) were considered unsafe. Moreover, the MOE to DON in cereals (605) is alarming, especially in bread (632) and manakesh (6879). The MOE to AFM1 in dairy products was 1454, indicating health-related risks with a focus on yogurt (9788) and labneh (8153). As for the herbs/spices group and traditional dishes, the MOE to AFB1 was relatively lower than 10,000 (3690 and 1625, respectively), with a focus on thyme (2624) and kishik (3297), respectively. It is noteworthy that the MOE to DON and the MOE to OTA in traditional foods and coffee were lower than 10,000 (8047 and 8867, respectively). All hazard quotient (HQ) values were below 1, except the HQ value of milk and dairy products (1.96). The intake of some food groups varied between age categories, corresponding to differences in EDI between them. Thus, it is essential to put control measures in place to decrease the contamination and exposure to mycotoxins by Lebanese consumers.

## 1. Introduction

Mycotoxins are low-molecular-weight secondary metabolites naturally produced by filamentous fungi, such as *Aspergillus*, *Penicillium* and *Fusarium* genera [1]. Mycotoxins are highly toxic to both animals and humans, causing adverse health effects at low concentrations and affecting various body sites and organs, ranging from acute to chronic, and potentially lethal at high doses [2]. Moreover, human exposure to mycotoxins mainly occurs through ingestion but can also happen through inhalation or contact. Oral exposure has been reported throughout human history and led to severe outbreaks, causing mycotoxins to be a source of concern in the food safety system [3]. Although there are more than 300 discovered mycotoxins, 30 of them are the most significant and commonly detected in food, including aflatoxins (AFs), ochratoxins (OTs), fumonisins (FBs), patulin, zearalenone (ZEN) and trichothecenes. These mycotoxins can naturally contaminate feed and food products, such as nuts, cereals, fruits, vegetables, spices, herbs and coffee beans, and can be detected in animal products, such as meat, milk and eggs, if the animals have consumed contaminated feed [4].

According to the United Nations Food and Agriculture Organization (FAO), contaminated food crops with mycotoxins constitute around 25% of crops worldwide [5]. This contamination occurs due to some environmental factors, such as moisture content, water activity, temperature, pH value, relative air humidity, degree of crop physical damage as well as the composition of the food matrix and the presence of mold spores [6,7]. Other factors encompass inappropriate agricultural, harvesting and storage practices, in addition to improper handling, processing or transportation [8].

Aflatoxins are among the most widespread mycotoxins that contaminate a variety of food products at different times in production [9]. They are produced by *Aspergillus* spp., mainly, *A. flavus*, *A. parasiticus* and *A. nomius*, at high temperature and humidity [10]. Aflatoxins can contaminate various food items, such as cereals, nuts, spices, herbs, oilseeds and legumes [11]. They are also acutely and chronically toxic to animals and humans, and their main adverse effect is known as aflatoxicosis, which is a disease caused by aflatoxin exposure [12,13]. Acute aflatoxicosis can result in abdominal pain, vomiting, diarrhea, pulmonary edema, anorexia, fatty liver, jaundice, depression and even death at very high doses [14]. Chronic aflatoxicosis that is caused by low levels of exposure over extended periods of time is associated with immune suppression and cancer, with the liver being the primary target organ in addition to other reproductive disorders [13]. Many aflatoxins have been identified (aflatoxin B, G and M), but aflatoxin B1 (AFB1) is the most frequent and toxic type. Aflatoxin M1 (AFM1) is a less toxic hydroxylated metabolite of AFB1, found in meat, eggs and dairy products from livestock that have ingested contaminated feed [15]. The International Agency for Research on Cancer (IARC) classified aflatoxins as group 1 known human carcinogens [16]. Ochratoxins are mycotoxins produced by *Aspergillus* and *Penicillium* species, particularly *A.ochraceus*, *A. carbonarius*, *A. circumdati*, *A. niger*, *P. verrucosum* and *P. nordicum*, and are present in three forms, ochratoxin A, B and C (OTA, OTB, OTC) [17]. The most common and toxic type is OTA, which is classified by the IARC as a possible human carcinogen (group 2B), with the main target organ being the kidney [18]. Many studies have shown that OTA can cause nephrotoxic, hepatotoxic, neurotoxic, teratogenic and immunotoxic effects [19]. OTA contaminates commodities, like cereal grains, wheat, fresh or dried fruits, coffee, oilseeds, tea, etc. [20]. Deoxynivalenol (DON) is a trichothecene commonly found in grains. It is known as vomitoxin because when agricultural animals consume it in high doses, it causes vomiting, nausea and diarrhea [21]. Chronic exposure to DON can lead to adverse health effects such as delayed growth and immunotoxic or hemotoxic effects [22]. Another important trichothecene produced by fusarium species is the T-2 toxin that is rapidly metabolized to HT-2 after ingestion. Acute intoxication with T-2 or HT-2 can cause severe health effects, such as vomiting, diarrhea, neuroendocrine changes and deterioration of the immune system, whereas chronic exposure leads to weight loss, stunting and reproductive defects [23]. To protect consumers from the negative health effects of mycotoxins and limit their exposure, several countries have adopted regulatory limits on food. However, regulatory levels are still lacking for some mycotoxins, such as T-2 and HT-2, and for other regulated ones, there are still missing levels for some food products such as aflatoxins in herbs [24]. Food products and crops in Lebanon are at a high risk of mycotoxin contamination due to inducive climatic conditions of high temperature and humidity, especially in summer. This creates a food safety and public health problem, specifically since mycotoxins are heat resistant and cannot be eliminated through subsequent processing steps, such as cooking, pasteurization or sterilization [25,26]. In Lebanon, protective measures such as regulatory limits adopted from the CODEX Alimentarius are in place to decrease consumers’ exposure. Thus, it is important to regularly monitor mycotoxin contamination in Lebanon and estimate the exposure level of Lebanese people to these mycotoxins. However, despite previous studies, data are scarce on this topic. Therefore, this study aimed to evaluate the dietary exposure of Lebanese adults to multi-mycotoxins (AFB1, AFM1, OTA, OTB, DON, T-2 and HT-2), assessing the associated health risks and comparing the health risks posed in different age categories.

## 2. Results

### 2.1. Sociodemographic Characteristics of the Participants

From the 449 participants recruited in this study, 59% were females and 41% were males, as shown in Table 1. The mean age ± SD of participants was 34.34 ± 12.80 years, with the majority (63.5%) being adults (25–59 years); 23.4% were young adults (20–24 years), 9.8% were older adolescents (18–19 years) and 3.3% were older adults (≥60 years). Participants were recruited from all Lebanese governorates as follows: Mount Lebanon (39.9%), North Lebanon (13.4%), South Lebanon (12.2%), Nabatieh (9.1%), Beirut (7.6%), Akkar (6.9%), Beqaa (6%) and Baalbeck-Hermel (4.9%). Around half of the participants (50.3%) were married, 45% were single and a few were divorced (2.7%) or widowed (2%). Further, 59.7% were educated to university level, 14.9% reached secondary school, 14.7% reached middle school, 10% reached primary school and 0.7% were not educated. Additionally, 49.7% of participants were not working at the time, with 68.7% being females and 22.3% being males. Regarding medical history, 74.2% of participants were healthy with no medical history, with the majority of them being males (81.5%) compared to females (69.1%). The overall mean height ± SD and weight ± SD were 165.28 ± 9.37 cm and 73.8 ± 17.05 Kg, respectively, with the majority of participants (38.5%) being overweight.

### 2.2. Food Consumption Pattern of Different Age Groups Attained from the National Consumption Survey

Table 2 presents the dietary intake of different age categories. The intake levels of the available age categories did not differ significantly for the majority of food groups, with the most consumed group by the study population being fruit and fruit products, with an average intake of 28.19 ± 31.59 g/day, followed by starchy roots and tubers (25.27 ± 21.67 g/day), vegetables and vegetable products (20.61 ± 11.73 g/day), sugar and confectionery (19.15 ± 22.30 g/day), milk and dairy products (15.35 ± 13.70 g/day) and cereals and cereal products (15.08 ± 6.40 g/day). On the other hand, the least consumed food groups were herbs, spices and condiments (5.98 ± 5.54 g/day); fish and other seafood (1.89 ± 3.49 g/day); fat and oils (1.44 ± 1.46 g/day); and, lastly, nuts and oilseeds (0.55 ± 1.38 g/day). Concerning beverages, water was the most consumed (1437.80 ± 840.23 mL/day), followed by stimulants (61.56 ± 48.93 mL/day) and alcoholic beverages (0.60 ± 5.87 mL/day). Meanwhile, the intake of traditional foods differed between the populations studied. Older adolescents and young adults had greater intake levels (8.72 ± 5.91 g/day and 10.74 ± 13.04 g/day, respectively) compared to adults who consumed 7.41 ± 7.14 g of traditional food daily. Similarly, young adults consumed 12.40 ± 13.11 g/day of legumes and pulses, less than the amount consumed by older adults (17.49 ± 8.36 g/day), whose intake exceeded that of adults (13.35 ± 10.27 g/day). Concerning fruit and vegetable juices, their dietary intake was 32.22 ± 41.57 g/day in young adults, far above the intake of older adults (12.92 ± 18.09 g/day) and adults (17.56 ± 25.28 g/day). Young adults also had a higher intake of meat and meat products (4.24 ± 3.36 g/day) compared to adults (3.70 ± 4.64 g/day). Moreover, older adolescents and young adults had an egg and product intake of 4.28 ± 10.15 g/day and 3.47 ± 4.21 g/day, respectively, much greater than that of adults (1.79 ± 2.07 g/day). Similarly, older adolescents had the highest consumption rate of snacks and desserts (4.28 ± 10.15 g/day), followed by young adults (3.47 ± 4.21 g/day), adults (1.79 ± 2.07 g/day) and older adults (1.06 ± 1.36 g/day). Finally, the intake of non-alcoholic beverages in older adults (22.86 ± 41.83 mL/day) was below that of older adolescents (39.49 ± 67.64 mL/day) and young adults (44.52 ± 73.78 mL/day).

### 2.3. Food Contamination Data, Dietary Exposure and Health-Related Risk Assessment from Each Food Group

#### 2.3.1. Cereals and Cereal Products

In cereals, AFB1 had an average concentration of 0.22 ± 0.292 µg/kg, less than the maximum tolerable limit (ML) set by the European Commission (EC) of 2 µg/kg for cereals and cereal products [27]. The food items that had the lowest and highest AFB1 concentrations were pasta (0.005 µg/kg) and rice (0.5 ± 0.30 µg/kg), respectively. Moreover, this food group had a total exposure to AFB1 of 0.86 ng/kg bw/day, with the food item contributing least to exposure being cornflakes (0.0003 ng/kg bw/day) and the one contributing most being rice (0.48 ng/kg bw/day). This exposure was associated with 0.0712 additional liver cancer cases/100,000 people/year. All MOE values were above 10,000, except for rice (827.90) and bulgur (4868.50) (Table 3). OTA had an average level of 0.843 ± 1.98 µg/kg, which is below the ML of 3 µg/kg set by the EC for cereals intended for direct human consumption [27]. The lowest concentration of OTA was found in pasta (0.18 µg/kg), while the highest was in bulgur (2.415 ± 6.605 µg/kg). Further, the exposure to OTA had a total of 3.29 ng/kg bw/day, with the food item contributing most being bread (1.18 ng/kg bw/day) compared to cornflakes (0.0006 ng/kg bw/day) that contributed the least. All MOE non-neo and MOE neo values were above 200 and 10,000, respectively. In addition, DON had a mean level of 89.08 µg/kg, with a range between 52 µg/kg in toast and 176 µg/kg in bread, smaller than the ML set by the EC for DON in cereals and cereal products of 750 µg/kg [27]. Moreover, the exposure to DON from cereals ranged from 0.11 ng/kg bw/day in cornflakes to 332.18 ng/kg bw/day in bread, with a total of 347.05 ng/kg bw/day. All MOE values were above 10,000, except for bread and manakeesh, which had values of 632.18 and 6879.87, respectively. Other mycotoxins (OTB, T-2 and HT-2) were also examined in bread in this study; however, they were below the respective LODs (limits of detection). This result was obtained from a study by Elaridi et al., who analyzed the mycotoxins in wheat grains, wheat flour and bread. No samples analyzed contained detectable levels of OTB, T-2 and HT-2 [28]. Thus, no health risk is posed to the consumer from the consumption of bread with respect to these mycotoxins.

#### 2.3.2. Fruit and Fruit Products

According to Table 3, the mean contamination level of AFB1 in fruit and fruit products was shown to be 0.22 µg/kg. The total intake of AFB1 was 0.03 ng/kg bw/day, which is associated with 0.0027 additional cancer cases/100,000 people/year, and the food item contributing most to the exposure was olives (0.024 ng/kg bw/day). The total MOE value (12,444.78) was above 10,000. The mean contamination of OTA was 0.08 µg/kg, and the exposure to OTA from this food group was 0.012 ng/kg bw/day, where olives had the highest exposure (0.009 ng/kg bw/day). MOE non-neo and neo were above 200 and 10,000, respectively. DON in fruit and fruit products had a mean of 62.5 µg/kg and an exposure of 9.13 ng/kg bw/day, with all MOE values above 10,000.

#### 2.3.3. Legumes and Pulses

The average concentration of OTA in legumes and pulses was 0.019 µg/kg, with peas, beans and green fava beans and green peas having the lowest concentration (0.01 µg/kg) and lentils having the highest concentration (0.05 µg/kg) (Table 3). The exposure to OTA from legumes and pulses was 0.017 ng/kg bw/day, with the highest-contributing food item being lentils (0.02 ng/kg bw/day) and the least-contributing being peas (0.0001 ng/kg bw/day). The total levels of MOE non-neo (275,071.74) and MOE neo (843,243.17) were above 200 and 10,000, respectively.

#### 2.3.4. Milk and Dairy Products

In milk and dairy products, AFM1 had a mean contamination level of 0.17 ± 0.397 µg/kg, greater than the ML of 0.05 µg/kg set by the EC for milk and dairy products (Table 3) [27]. The highest concentration of AFM1 was found in karicheh (0.8282 µg/kg) compared to milk that had the lowest (0.0225 µg/kg). Further, the total exposure to AFM1 was 0.39 ng/kg bw/day, where labneh was the highest-contributing food item (0.07 ng/kg bw/day), and this exposure is associated with 0.003 additional liver cancer cases/100,000 people/year. All MOE values were above 10,000, except for labneh (8153.18) and yogurt (9788.10).

#### 2.3.5. Herbs, Spices and Condiments

In herbs, spices and condiments, the average level of AFB1 was 0.37 ± 0.74 µg/kg, less than the maximum limit set by the Lebanese Standards Institution (LIBNOR) for thyme and thyme mixes (2 µg/kg) and the ML set by the EC for spices (5 µg/kg) [27,29]. Seeds had the lowest AFB1 concentration (0.22 µg/kg), and thyme had the highest (0.52 ± 0.74 µg/kg). The total exposure to AFB1 was 0.108 ng/kg bw/day, associated with 0.009 additional liver cancer cases/100,000 persons/year. All MOE values were above 10,000, except for thyme (2624.32).

The OTA ranged between 0 and 0.08 µg/kg in thyme and seeds, respectively, with an average of 0.04 µg/kg, which is below the maximum limit set by LIBNOR for thyme and thyme mixes of 3 µg/kg and the ML set by the EC for OTA in spices of 15–20 µg/kg [27,29]. In addition, the total exposure level to OTA reached a value of 0.012 ng/kg bw/day, with seeds contributing most to the exposure (0.001 ng/kg bw/day). MOE non-neo (403,694.19) and MOE neo (1,237,540.32) were above 200 and 10,000, respectively. DON was only analyzed in seeds and had an average concentration of 62.5 µg/kg. The total exposure to DON from herbs, spices and condiments was 18.31 ng/kg bw/day, which was associated with a total MOE of 11,470.72, greater than 10,000. Please refer to Table 4.

#### 2.3.6. Snacks and Desserts

Table 4 shows that AFB1 had an average concentration of 0.11 µg/kg in snacks and desserts, where pastries were the only food item analyzed. The total exposure to AFB1 was 0.018 ng/kg bw/day, which is associated with 0.0015 additional liver cancer cases/100,000 persons/year. In addition, the total MOE value (22,054.64) was above 10,000. On the other hand, the average OTA was 0.364 µg/kg, where chocolate had the lowest concentration (0.025 µg/kg) and biscuits (0.71 µg/kg) had the highest. OTA also had a total exposure of 0.06 ng/kg bw/day, MOE non-neo greater than 200 (78,775.96) and MOE neo above 10,000 (241,490.79). Concerning DON, its average concentration was 62.134 µg/kg, lower than the ML set by the EC of 500 µg/kg for some desserts and snacks, particularly pastries, biscuits and cereal snacks [27]. The food item with the lowest concentration was biscuits (31 µg/kg), while the one with the highest was pastries (109.67 µg/kg). Moreover, the exposure to DON from snacks and desserts was 10.24 ng/kg bw/day, and MOE had a total of 20,498.53, which is above 10,000.

#### 2.3.7. Traditional Food

According to Table 4, traditional food had a mean AFB1 level of 0.83 ± 0.44 µg/kg and an exposure of 0.25 ng/kg bw/day, associated with 0.02 additional liver cancer cases/100,000 people/year. This food group had an alarming MOE value (1625.16) below 10,000, particularly for kishik. The contamination level of OTA ranged between 0.025 µg/kg in kibbeh and 1.14 µg/kg in kishik, with an average of 0.56 µg/kg. The total EDI of OTA was 0.166 ng/kg bw/day; the item contributing most to this exposure was kishik (0.167 ng/kg bw/day) compared to kibbeh with the lowest exposure (0.003 ng/kg bw/day). All MOE values were above 10,000.

Concerning DON, its contamination level in traditional food was 88 µg/kg. Moreover, the exposure to DON was 26.10 ng/kg bw/day, where the main source of DON intake was meat pies, and the total MOE (8047.34) was below 10,000.

#### 2.3.8. Alcoholic Beverages and Stimulants

Referring to Table 4, the average level of OTA in alcoholic beverages was 1.47 µg/kg, below but close to the ML set by European legislation for OTA in wine (2 µg/kg) [27]. The average exposure to OTA was 0.01 ng/kg bw/day, MOE non-neo (468,475.62) was above 200 and MOE neo (1,436,130.33) was above 10,000.

Moreover, in alcoholic beverages, DON had a mean of 52.08 µg/kg, an exposure level of 0.36 ng/kg bw/day and an MOE of 587,072.19, greater than 10,000.

On the other hand, in stimulants, the average concentration of OTA was 0.51 µg/kg, which is less than the ML set by the EC of 10 µg/kg for soluble coffee [27]. The dietary intake of OTA from stimulants was 0.53 ng/kg bw/day, MOE non-neo was 8867.94 and MOE neo was 27,185.01, where they were above 200 and 10,000, respectively.

#### 2.3.9. Nuts and Oilseeds

In nuts and oilseeds, AFB1 had a mean concentration of 0.4 ± 0.296 µg/kg, far below the ML of 2 µg/kg set by the EC for groundnuts, nuts and processed products intended for direct human consumption or use as an ingredient in foodstuffs [27]. The exposure to AFB1 from nuts was 0.0025 ng/kg bw/day, associated with 0.0385 additional liver cancer cases/100,000 people/year. All MOE values were above 10,000. Similarly, OTA had an average concentration of 0.25 µg/kg, as presented in Table 4, an exposure of 0.002 ng/kg bw/day, MOE non-neo (2,974,696.01) greater than 200 and MOE neo (9,119,046.98) above 10,000.

#### 2.3.10. Fats and Oils

Although oils were investigated in this study for the presence of AFB1, the mean contamination level was 0 µg/kg. This mean was obtained from the total diet study conducted by Raad et al. in 2014, where AFB1 was not detected in oils when taking into account the lower-bound estimate level of 0 µg/kg [13].

#### 2.3.11. Calculation of HQ for All Food Groups

Concerning HQ related to the multi-mycotoxins examined in this study, all food groups had values below 1, except for milk and dairy products that had a total HQ value of 1.96, which indicates that the consumption of milk and dairy products is associated with a non-tolerable exposure level to AFM1. Regarding the weekly exposure to OTA, all food groups had values below the PTWI set by JECFA for OTA of 100 ng/kg bw [30] (Table 3 and Table 4).

### 2.4. Total Dietary Exposure to Mycotoxins (AFB1, AFM1, OTA and DON) and Its Associated Risks Related to the Consumption of Food Groups

- AFB1

According to Table 5, the total exposure to AFB1 was 1.26 ng/kg bw/day, and the food group contributing most to the exposure was cereals and cereal-based products (68%). Traditional foods as well as herbs, spices and condiments were other important food sources for AFB1, contributing 19.5% and 8.6% to the daily exposure, respectively. Last, the nuts and oilseeds food group (0.2%) contributed less than 1% to total exposure. The total exposure to AFB1 could be associated with 0.105 additional liver cancer cases/100,000 persons/year. Moreover, all HQ values were shown to be less than 1, and MOE values were above 10,000 for most food groups, except for cereals (466.59), traditional food (1625.16) as well as herbs spices and condiments (3690.71).

ii. AFM1

In this study, the total exposure to AFM1 was 0.39 ng/kg bw/day, with milk and dairy products being the only food group contributing to this exposure (100%). This exposure is accompanied by an additional 0.003 liver cancer cases/100,000 persons/year. Further, the total HQ value (1.96) was greater than 1, and MOE had a total (1454.06) less than 10,000, indicating a major risk of exposure to AFM1.

iii. OTA

The total exposure to OTA was 4.10 ng/kg bw/day, with cereals (80%) being the food group contributing most to the total exposure and nuts and oilseeds (0.04%) being the least-contributing group. In addition, the overall weekly exposure was 28.68 ng/kg bw, less than the PTWI set by JECFA for OTA of 100 ng/kg bw. Concerning HQ, all food groups had values below 1. MOE non-neo had a value of 1154.45, which is greater than 200, but MOE neo had a concerning value (3539) below 10,000, with cereals being the food group contributing to this alarming MOE value.

iv. DON

The total exposure to DON was found to be 411.18 ng/kg bw/day, with the food groups contributing most to the exposure being cereals and cereal products (84.4%) compared to alcoholic beverages, the least contributing (0.1%) (Table 5). The overall HQ (0.0514) was far below 1, and the overall MOE (510.72) was below 10,000, indicating a major exposure to DON, particularly from cereals (605.11) and traditional food (8047.34).

### 2.5. Total Estimated Dietary Exposure and Risk Assessment per Age Groups

When comparing the estimated daily intake of AFB1, AFM1, OTA and DON for different age groups in Lebanon, we deduce that DON, which had the highest total exposure in our study, demonstrated the highest EDI average in all age groups when compared to other mycotoxins, as shown in Table 6. The highest EDI of DON was reported for older adolescents, with a value of 493.73 ng/kg bw/day, followed by young adults (480.33 ng/kg bw/day), adults (393.70 ng/kg bw/day) and older adults (356.59 ng/kg bw/day). Meanwhile, the EDI of AFM1 was approximately similar in all age groups, with values ranging between 0.16 and 0.17 ng/kg bw/day and older adults being slightly more exposed to AFM1 than other age groups. Still, the EDI values of AFM1 were lower than the EDI of AFB1 and OTA in the different age groups. For instance, the intake of AFB1 in older adolescents (1.05 ng/kg bw/day) was close to that of young adults (1.06 ng/kg bw/day) but higher than that of adults (0.89 ng/kg bw/day) and older adults (0.79 ng/kg bw/day). While the EDI of OTA in older adolescents (4.05 ng/kg bw/day) was lower than that of young adults (4.15 ng/kg bw/day), both were higher than that of adults (3.40 ng/kg bw/day) and older adults (3.23 ng/kg bw/day). Thus, it is evident that younger age groups are more susceptible to mycotoxin exposure than older ones. Moreover, all age groups had HQ values below 1. All MOE values were below 10,000 for all age groups, which is alarming. When comparing the risk of liver cancer from AFB1 and AFM1, we conclude that AFB1 was associated with the highest risk of liver cancer among the age categories, where older adolescents and young adults had the highest risk of 0.09 additional cancer cases/100,000 people/year.

## 3. Discussion

In this study, all food groups had contamination levels below the ML set by the EC, except for milk and dairy products, which had an AFM1 concentration of 0.17 ± 0.397 µg/kg exceeding the ML (0.05 µg/kg). The total exposure to AFB1, AFM1, OTA and DON was 1.26, 0.39, 4.10 and 411.18 ng/kg bw/day, respectively. AFB1 and AFM1 were associated with 0.105 and 0.003 additional cancer cases/100,000 persons/year, respectively, while OTA had a weekly exposure of 28.68 ng/kg bw less than the PTWI of 100 ng/kg bw. Almost all MOE calculations were above 10,000, except MOE to AFB1 for cereals (466.59), traditional food (1625.16) and herbs (3690.71); to AFM1 for milk and dairy products (1454.06); to OTA for cereals (4412.76); and to DON for cereals (605.11) and traditional food (8047.34). All HQ values were below 1, except for milk and dairy products (1.96), and the intake of some food groups varied between age categories, corresponding to the difference in exposure levels between them.

### 3.1. Comparison with National Studies

The exposure to AFB1 in this study was 1.26 ng/kg bw/day, greater than the exposure reported by Raad et al. in the total diet study for regular consumers (0.63–0.66 ng/kg bw/day) but lower than that reported for excessive consumers (1.40–1.46 ng/kg bw/day for) [15]. It was also greater than that reported by Daou et al. for wheat and wheat products (0.92 ng/kg bw/day) [11]. Consequently, the exposure to AFB1 in this study was associated with 0.105 additional liver cancer cases/100,000 people/year, greater than the risk observed in studies by Raad et al. of 0.053–0.055 additional liver cancer cases/100,000 people/year for regular consumers [15] and Daou et al. (0.076 HCC/100,000 people/year) [11] but less than that reported Hassan et al. (0.35–0.41 HCC/100,000 people/year) [31]. OTA had an exposure of 4.10 ng/kg bw/day, higher than the exposure level reported in other Lebanese studies, such as one conducted by Hassan et al. (1.345 ng/kg bw/day) [31]; however, it was lower than that reported by Raad et al. (4.28 ng/kg bw/day) [15] and Daou et al. (7.60 ng/kg bw/day) [11]. For AFM1, the total exposure (0.39 ng/kg bw/day) was greater than the level obtained from a study by Hassan et al. (0.14 ng/kg bw/day) [32]. Finally, the exposure level to DON in this study (411.18 ng/kg bw/day) was greater than that obtained from a study by Raad et al. in 2005 (190 ng/kg bw/day) but lower than the value obtained from his study in 2014 (1560 ng/kg bw/day) [15,33]. The differences seen between this study and other studies might be due to differences in sample types, methods of testing, analysis and in the ways contamination data were generated.

### 3.2. Comparison with International Studies

The total exposure to AFB1 in this study (1.26 ng/kg bw/day) was higher than the exposure reported in several other countries, including The Netherlands (0.1 ng/kg bw/day) [34], France (0.002–0.22 ng/kg bw/day) [35], Canada (1 ng/kg bw/day) [36] and Korea (1.19 ng/kg bw/day) [37]. In our study, the main contributor to the exposure to AFB1 was cereals (68%), compared to rice (98.96%) in Korea [37] and chocolate in France [35], whereas AFM1 had a total exposure of 0.39 ng/kg bw/day in this study, greater than the levels reported in Brazil (0.188 ng/kg bw/day) [38], The Netherlands (0.19 ng/kg bw/day) [34], France (0.03 ng/kg bw/day) [35] and Ireland (0.0093 ng/kg bw/day) [39]. For OTA, the total exposure was reported to be 4.10 ng/kg bw/day, greater than that reported in Ireland (0.000049 ng/kg bw/day) [39], the United Kingdom (0.53 ng/kg bw/day) [40], Italy (1.13 ng/kg bw/day) [41] and Spain (1.18 ng/kg bw/day) [42]. The main contributor to this exposure level was cereals (80%) in our study, unlike the result obtained in France, where coffee (38.1%) was noted as the main contributor to the total exposure to OTA [43]. Meanwhile, the total exposure to DON in this study was 411.18 ng/kg bw/day, much higher than that reported in the United Kingdom (0.17 ng/kg bw/day) [40], Ireland (0.20 ng/kg bw/day) [39], The Netherlands (0.34 ng/kg bw/day) [34] and France (0.373–0.379 ng/kg bw/day) [43]. The main contributor to the exposure to DON in this study was cereals (84.4%), which was also reported as a main contributor in other countries, including France (90%) [43] and The Netherlands (52.30%) [34].

## 4. Strengths and Limitations

This is the first study of its kind in the Middle East and North Africa to investigate the presence of multi-mycotoxins in a wide variety of food items, particularly 38 food items, and to estimate the exposure level of the Lebanese population to these mycotoxins as well as to assess all risk characterization components (HQ, MOE, liver cancer cases, kidney disease risk) for the whole population and for different age categories to be compared. Despite that, the current study featured to some limitations. Initially, due to the absence of funding for this project, we used previous contamination data found in the Lebanese literature review. Additionally, the method used to obtain consumption data (FFQ) depended on memory, meaning that participants might under- or overestimate their dietary intake. In addition, there are other food items that contribute to mycotoxin exposure, which were not analyzed in this study. Thus, future studies should test food items in the laboratory using appropriate methods to evaluate mycotoxin contamination levels that will provide more accurate results on the occurrence and exposure trends to mycotoxins in Lebanon. Also, they should consider other food items that contribute to the exposure.

## 5. Conclusions

In conclusion, this study investigated the contamination of several food items consumed in Lebanon with multi-mycotoxins (AFB1, AFM1, OTA, OTB, DON, T-2 and HT-2), the exposure of the Lebanese population to these mycotoxins as well as the risk posed to consumers’ health. We deduced that, except for milk and dairy products, all food groups had contamination levels below the ML. Some food groups (cereals and cereal products; herb spices and condiments; milk and dairy products and traditional food) had alarming MOE values that showed an increased risk of exposure to certain mycotoxins. Moreover, the HQ value of milk and milk products (1.96) was above 1, and all total MOE values were below 10,000 (except MOE non-neo of OTA, which was above the limit of 200), indicating that the Lebanese population is at a high risk of exposure to mycotoxins, with younger age groups (older adolescents and young adults) being the most susceptible. Thus, it is crucial to put control measures in place to lessen the presence of mycotoxins and the exposure of Lebanese people to them. Some measures include regular monitoring and inspections on facilities that store crops and main staple food items to make sure they are being stored appropriately and that the facilities are complying with the regulations. Strict regulations should be put in place to prevent farmers from feeding animals contaminated feed that contribute to the production of AFM1 in milk and other animal products. Finally, more research on the presence of mycotoxins in food should be conducted in Lebanon to stay informed on contamination patterns over time, changes in exposure levels and to ensure that the upper limits are not being surpassed.

## 6. Materials and Methods

### 6.1. Food Consumption Data

#### 6.1.1. Study Design and Participants’ Recruitment

A cross-sectional survey was conducted over a 5-month period from May to September 2022. The clusters from where participants were recruited were eight Lebanese governorates (Beirut, Mount Lebanon, North Lebanon, Akkar, South Lebanon, Nabatieh, Beqaa and Baalbeck-Hermel). Probability proportional to size sampling method was used to recruit participants from each district, and in order to determine the representative sample size, a single population formula was used, n = [p (1 − p)] × [(Z∝/2)2/(e)2], where n represents the sample size, Z∝/2 is the reliability coefficient of standard error at a 5% level of significance = 1.96, p represents the probability of adults (18–64 years) who were unable to practice preventive measures of the diseases (50%) and e represents the level of standard error tolerated (5%), as stated by Hosmer and Lemeshow [44]. Based on this formula, the minimum representative sample size sufficient to ensure appropriate power for statistical analyses is 400 participants. Considering a 10% non-response rate, we reached a total of 449 Lebanese adult participants.

#### 6.1.2. Inclusion and Exclusion Criteria

**Figure 1:**
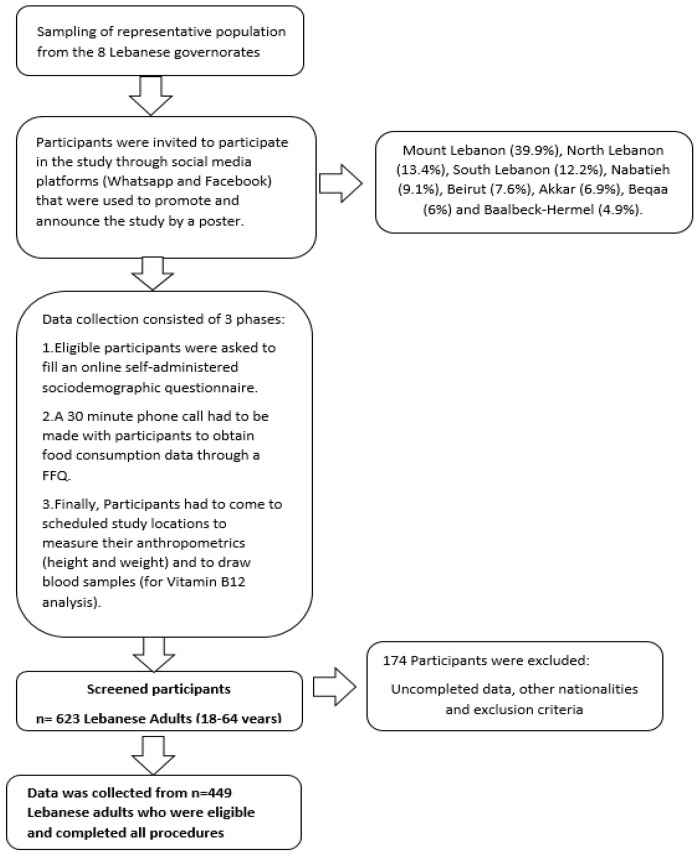
Flow chart of the recruitment process.

To be included in the study, participants had to be Lebanese, aged between 18 and 64 years and either gender. We reached out to 2 participants per household and tried to equalize the number of participants from both genders. The recruitment process is presented in Figure 1.

#### 6.1.3. Study Instruments

A sociodemographic questionnaire was used to obtain information about participants’ age, residency, educational and income level, marital and occupation status, and medical history. Moreover, a 24 h recall and a validated 157-item semi-quantitative FFQ were used as part of the national consumption survey in a 30 min interview with participants to obtain information about their dietary intake of various food groups in the past 6 months [45]. Food consumption data were recorded as daily, weekly, and monthly, then transformed to g/day. Anthropometric data were also collected.

#### 6.1.4. Ethical Approval

The research/ethical committee at Al Zahraa University Medical Center approved the research design, protocol, and implementation (Ethics code: #231/12 January 2022).

### 6.2. Food Contamination Data

Food contamination data were obtained from the Lebanese mycotoxin book [25], which is a compilation of all studies published in Lebanon between 2004 and 2022. The food’s mean, standard deviation (SD) and minimum and maximum contamination value were collected to assess dietary exposure and the associated health risks. Table 7 shows the type of mycotoxins analyzed in the food groups consumed by participants according to WHO GEMS distribution.

#### 6.2.1. Calculation of Dietary Exposure

The dietary exposure for each participant was calculated by multiplying the mean mycotoxin contamination level for a certain food item by the dietary intake of the same food item obtained in the current study, then dividing by the participants’ body weight. The equation is as follows:

$$EDI\;(ng/kg\;bw/day)=\frac{DI\;\left(\frac{kg}{day}\right)\times MC\;(ng/kg)}{Body\;Weight\;(kg)}$$

where EDI is the estimated daily intake, MC is the mean concentration taken from the Lebanese book [25] and DI is the daily intake of each food item. After calculating the EDI for every participant, the average daily exposure for each mycotoxin was calculated for every food group by adding the exposure of all participants and dividing it by the sample size of 449. The average daily exposure to each mycotoxin from every item was summed to obtain the total daily exposure (ng/kg bw/day).

#### 6.2.2. Validation and Quality Assurance Methods

Data were obtained from studies that mainly used methods of high-performance liquid chromatography (HPLC), liquid chromatography–mass spectrometry (LC-MS), ultra-performance liquid chromatography–mass spectrometry (UPLC-MS) and enzyme-linked immunosorbent assay (ELISA). The limits of detection and limits of quantification were obtained from each study and are represented as ranges in Table 8 for each mycotoxin in different food groups studied. The data were included from various studies after checking their quality measures including recovery rates, which are also presented as ranges in Table 8 for each mycotoxin in the different food groups.

### 6.3. Risk Assessment and Characterization

#### 6.3.1. Margin of Exposure Calculation

Margin of exposure (MOE) approach established by the European Food Safety Authority (EFSA) [46] represents the margin between a dose and exposure causing cancer in animals or humans, with the estimated human exposure to the substance, calculated as follows:

$$MOE=\frac{BMDL10\;(ng/kg\;bw/day)}{EDI\;(ng/kg\;bw/day)}$$

where BMDL10 is the benchmark dose lower confidence limit, and EDI is the estimated daily intake. The BMDL10 values used for AFB1 and AFM1 were 0.4 μg/kg bw/day (EFSA) [46] and 570 ng/kg bw/day (Sharafi et al.) [47], respectively. For OTA, EFSA established two BMDL10 values, one for critical neoplastic effects (14.5 μg/kg bw/day) and one for chronic non-neoplastic effects (4.73 μg/kg-bw/day) [48]. For DON, a BMDL10 of 0.21 mg/kg bw/day was used (EFSA) [49]. Since T-2 and HT-2 had means of 0 µg/kg, we did not calculate the risk assessment components for them. For OTA, MOE neoplastic (MOE neo) ≥ 10,000 or MOE non-neoplastic (MOE non-neo) ≥ 200 indicates that the exposure is of low health concern [48]. For other mycotoxins, MOE ≥ 10,000 indicates low risk, while MOE < 10,000 indicates a major risk [50].

#### 6.3.2. Hazard Quotient Calculation

According to EFSA, hazard quotient (HQ) is the ratio of the potential exposure to the substance to the level at which no adverse effect is expected, such as tolerable daily intake (TDI) or acute reference dose (ARfD) [50]. The equation used is as follows:

$$HQ=\frac{EDI\;(ng/kg\;bw/day)}{TDI\;or\;ARfD\;(ng/kg\;bw/day)}$$

For AFB1, AFM1, OTA and DON, the reference values used to calculate HQ were 0.017–0.082 μg/kg bw/day (Turna and Wu) [51], 0.2 ng/kg bw/day (Sharafi et al.) [47], 18 ng/kg bw/day (ESFA) [52] and 8 μg/kg bw/day (Joint FAO/WHO Expert Committee on Food Additives) [53], respectively. If HQ is less than 1, this indicates tolerable exposure, and if it is a greater than 1, this indicates a non-tolerable exposure level [50].

#### 6.3.3. Liver Cancer Risk for AFB1 and AFM1

According to JECFA, it is estimated that for non-European countries, the ingestion of 1 ng/kg bw/day of AFB1 and AFM1 induces 0.083 and 0.0083 liver cancer cases or hepatocellular carcinoma (HCC)/100,000 persons/year, respectively [54]. So, the liver cancer risk based on the exposure to AFB1 and AFM1 (ng/kg bw/day) can be calculated as:

$$Liver\;cancer\;risk\;AFB1=\frac{Exposure\;of\;AFB1\;(ng/kg\;bw/day)\times 0.083\;cancer\;cases/100,000\;persons}{1\;(ng/kg\;bw/day)}$$

$$Liver\;cancer\;risk\;AFM1=\frac{Exposure\;of\;AFM1\;(ng/kg\;bw/day)\times 0.0083\;cancer\;cases/100,000\;persons}{1\;(ng/kg\;bw/day)}$$

#### 6.3.4. Weekly Exposure to OTA

In order to evaluate the risk of kidney disease, we calculated the weekly exposure to OTA and compared it to the provisional tolerable weekly intake (PTWI) set by JECFA of 100 ng/kg bw, which was taken into consideration [33].

## 7. Statistical Analysis

The data were analyzed using Microsoft Excel 2013 and IBM SPSS statistics Version 24 software. Excel was used to transform consumption data from daily, weekly and monthly frequencies to g/day for each participant. Similarly, EDI and risk assessment components were performed for each participant on Excel, and the mean was calculated for each component. Population characteristics were presented as frequencies and percentages (categorical variables) or as mean and SD (continuous variables). These characteristics were analyzed for the whole study population and according to gender. The Mann–Whitney U test was used to check if the mean of continuous variables differed based on gender. Chi-Square test and Fisher’s exact test were used to assess differences in categorical variables between males and females. The Kruskal–Wallis test was carried out to examine the differences in dietary intake levels between the different age categories, together with the Mann–Whitney U test, which was used to explain a significant effect. A *p*-value < 0.05 was considered significant for all analytical tests.

## Figures and Tables

**Table 1:** General demographic characteristics of the study population.

	Overall (n = 449, 100%)		Females (n = 265, 59%)		Males (n = 184, 41%)		
	Mean ± SD		Mean ± SD		Mean ± SD		*p*-Value
Age	34.34 ± 12.80		34.35 ± 12.58		34.33 ± 13.16		0.812
Height	165.28 ± 9.37		159.54 ± 5.85		173.54 ± 7.03		<0.0001 **
Weight	73.8 ± 17.05		68.20 ± 15.04		81.84 ± 16.61		<0.0001 **
BMI	26.99 ± 5.78		26.86 ± 6.12		27.16 ± 5.26		0.327
	Variables analyzed by Mann–Whitney U test						
	** significant at *p*-value < 0.0001 for Mann–Whitney U test.						
	N	%	n	%	n	%	*p*-Value
Age							0.099 ^a^
Older adolescents (18–19 years)	44	9.8	22	8.3	22	12.0	
Young adults (20–24 years)	105	23.4	65	24.5	40	21.7	
Adults (25–59 years)	285	63.5	173	65.3	112	60.9	
Older adults (60–65 years)	15	3.3	5	1.9	10	5.4	
BMI							0.127 ^b^
Underweight	19	4.2	15	5.7	4	2.2	
Normal weight	151	33.6	94	35.5	57	31.0	
Overweight	173	38.5	93	35.1	80	43.5	
Obese	106	23.6	63	23.8	43	23.4	
Residence							0.007 ^a^ *
Akkar	31	6.9	20	7.5	11	6.0	
Baalbeck	22	4.9	12	4.5	10	5.4	
Beqaa	27	6.0	22	8.3	5	2.7	
Beirut	34	7.6	24	9.1	10	5.4	
Mount lebanon	179	39.9	106	40.0	73	39.7	
Nabatieh	41	9.1	29	10.9	12	6.5	
North lebanon	60	13.4	27	10.2	33	17.9	
South Lebanon	55	12.2	25	9.4	30	16.3	
Marital Status							0.052 ^b^
Single	202	45.0	121	45.7	81	44.0	
Married	226	50.3	128	48.3	98	53.3	
Divorced	12	2.7	7	2.6	5	2.7	
Widowed	9	2.0	9	3.4	0	0.0	
Educational Level							0.567 ^b^
Did not attend school	3	0.7	2	0.8	1	0.5	
Primary	45	10.0	23	8.7	22	12.0	
Middle	66	14.7	44	16.6	22	12.0	
Secondary	67	14.9	39	14.7	28	15.2	
University	268	59.7	157	59.2	111	60.3	
Current Occupation							<0.0001 ^a^ **
Not working	223	49.7	182	68.7	41	22.3	
Full time	121	26.9	48	18.1	73	39.7	
Part time	43	9.6	21	7.9	22	12.0	
Self-employed	62	13.8	14	5.3	48	26.1	
Medical History							0.003 ^a^ *
No diseases	333	74.2	183	69.1	150	81.5	
Have disease	116	25.8	82	30.9	34	18.5	

* Significant at *p*-value < 0.05 for χ^2^ test; ** Significant at *p*-value < 0.0001 for χ^2^ test. ^a^ Variables analyzed by Chi square test. ^b^ Variables analyzed by Fisher test.

**Table 2:** Dietary intake of food groups obtained from the National Consumption Survey and divided according to age groups.

	Mean Dietary Intake ± SD (g/day)					
Food Groups Based on GEMS *	Overall Population (n = 449)	Older Adolescents (18–19 years) n = 44	Young Adults (20–24 years) n = 105	Adults (25–59 years) n = 285	Older Adults (≥60 years) n = 15	*p*-Value
Lebanese food basket items						
Cereals and cereal products	15.08 ± 6.40	15.33 ± 5.6	16.47 ± 7.25	14.59 ± 6.18	14.03 ± 5.29	0.169
Traditional food	8.36 ± 8.83	8.72 ± 5.91	10.74 ± 13.04	7.41 ± 7.14	8.67 ± 5.44	0.006 *
Starchy roots and tubers	25.27 ± 21.67	23.78 ± 14.31	29.24 ± 27.56	23.71 ± 19.04	31.48 ± 34.48	0.438
Nuts and oilseeds	0.55 ± 1.38	0.49 ± 0.72	0.81 ± 2.35	0.46 ± 0.88	0.70 ±1.37	0.231
Legumes and pulses	13.36 ± 10.90	14.29 ± 9.69	12.40 ± 13.11	13.35 ± 10.27	17.49 ± 8.36	0.04 *
Vegetables and vegetable products	20.61 ± 11.73	20.10 ± 10.23	22.43 ± 12.68	19.72 ± 11.17	26.12 ± 16.83	0.123
Milk and dairy products	15.35 ± 13.70	15.77 ± 15.30	15.89 ± 14.99	14.90 ± 13.08	18.72 ± 11.05	0.275
Fruit and fruit products	28.19 ± 31.59	23.33 ± 20.90	30.55 ± 33.35	28.28 ±32.89	24.33 ± 15.86	0.856
Fruit and vegetable juices	21.89 ± 31.91	28.35 ± 40.60	32.22 ± 41.57	17.56 ± 25.28	12.92 ± 18.09	0.001 *
Herbs, spices and condiments	5.98 ± 5.54	5.7 ± 5.55	6.19 ± 5.95	6.00 ± 5.44	4.82 ± 4.69	0.793
Meat and meat products	3.83 ± 4.16	3.92 ± 2.76	4.24 ± 3.36	3.70 ± 4.64	3.26 ± 2.76	0.01 *
Eggs and egg products	22.08 ± 32.49	29.15 ± 36.31	25.17 ± 31.07	20.28 ± 32.96	13.83 ± 12.43	0.032 *
Fish and other seafood	1.89 ± 3.49	2.69 ± 7.07	1.98 ± 2.79	1.73 ± 2.90	1.98 ± 2.12	0.199
Sugar and confectionery	19.15 ± 22.30	22.15 ± 29.40	20.58 ± 22.74	18.53 ± 21.23	12.05 ± 12.96	0.511
Desserts and snack	2.40 ± 4.21	4.28 ± 10.15	3.47 ± 4.21	1.79 ± 2.07	1.06 ± 1.36	<0.0001 **
Fat and oils of animals and vegetables	1.44 ± 1.46	1.53 ± 1.46	1.23 ± 1.174	1.49 ± 1.56	1.63 ± 1.11	0.257
Drinking water ^a^	1437.80 ± 840.23	1223.18 ± 729.38	1358.86 ± 753.36	1493.17 ± 889.52	1568 ± 646.96	0.118
Stimulant beverages ^a^	61.56 ± 48.93	50.73 ± 38.94	59.82 ± 44.50	63.73 ± 52.42	64.38 ± 31.54	0.401
Non-alcoholic beverages ^a^	34.97 ± 60.22	39.49 ± 67.64	44.52 ± 73.78	31.39 ± 53.77	22.86 ± 41.83	0.04 *
Alcoholic beverages ^a^	0.60 ± 5.87	0	0.08 ± 0.77	0.87 ± 7.31	0.80 ± 3.10	0.315

^a^ Beverages are presented in ml/day. * Significant at *p*-value < 0.05 for Kruskal–Wallis test; ** significant at *p*-value < 0.0001 for Kruskal–Wallis test.

**Table 3:** Dietary intake, EDI of analyzed mycotoxins and risk assessment components (MOE, HQ, liver cancer cases, weekly exposure) in main food groups including cereals; legumes and pulses; fruits and fruit products; and milk and dairy products.

			Mycotoxins																				
			AFB1						OTA						DON				AFM1				
Food Groups	Food Items	Mean Dietary Intake (g/day)	Mean AFB1 (µg/kg)	EDI (ng/kg bw/day)	HQ Lower Limit	HQ Upper Limit	MOE	Liver Cancer Risk (Cancer Cases/100,000 Persons)	Mean OTA (µg/kg)	EDI (ng/kg bw/day)	HQ	MOE non-neo	MOE neo	Weekly Exposure (ng/kg bw)	Mean DON (µg/kg)	EDI (ng/kg bw/day)	HQ	MOE	Mean AFM1 (µg/kg)	EDI (ng/kg bw/day)	HQ	MOE	Liver Cancer Risk (Cancer Cases/100,000 Persons)
Cereals and cereal based products	Bread	136.42	0.01	0.02	0.0002	0.0011	21,193.14	0.0016	0.623	1.18	0.0653	4022.61	12,331.48	8.23	176	332.18	0.0415	632.18	_				
	Baguette	3.29	0.2	0.01	0.0001	0.0006	41,377.68	0.0008	0.2	0.01	0.0005	489,291.05	1,499,940.86	0.07	_								
	Toast	0.48	0.28	0.002	0.00003	0.0001	184,485.14	0.0002	2.38	0.02	0.0010	256,651.38	786,774.85	0.13	52	0.40	0.0001	521,525.29					
	Kaak	3.61	0.46	0.02	0.0003	0.0015	16,098.98	0.0021	1.33	0.07	0.0040	65,842.39	201,842.43	0.50	70	3.78	0.0005	55,541.47					
	Cornflakes	0.13	0.16	0.0003	0.000004	0.00002	1,275,809.05	0.00003	0.33	0.0006	0.00004	7,314,638.55	22,423,310.56	0.005	58	0.11	0.00001	1,847,723.45					
	Rice	67.44	0.5	0.48	0.0059	0.0284	827.90	0.0401	0.645	0.62	0.0346	7589.06	23,264.57	4.36	_								
	Bulgur	18.35	0.32	0.08	0.0010	0.0048	4868.50	0.0068	2.415	0.62	0.0344	7628.33	23,384.95	4.34									
	Pasta	21.18	0.005	0.002	0.00002	0.0001	258,803.44	0.0001	0.18	0.06	0.0031	85,009.74	260,600.69	0.39	62.5	19.32	0.0024	10,869.74					
	Pies	2.40	0.0455	0.002	0.00002	0.0001	265,904.36	0.0001	0.22	0.007	0.0004	650,302.35	1,993,527.30	0.05	121.16	4.01	0.0005	52,424.81					
	Pizza	9.66	_						0.51	0.07	0.0038	68,734.04	210,706.90	0.48	85	11.47	0.0014	18,309.70					
	Manakesh	24.53							0.445	0.15	0.0086	30,643.94	93,940.20	1.08	88	30.52	0.0038	6879.87					
Total *		287.47	0.22	0.86	0.0105	0.0504	466.59	0.0712	0.843	3.29	0.183	1439.47	4412.76	23.00	89.08	347.05	0.0434	605.11					
Fruit and Fruit products	Dried Fruits	3.10	0.22	0.01	0.0001	0.0006	39,359.16	0.0008	0.08	0.004	0.0002	1,279,910.70	3,923,616.30	0.03	62.5	2.89	0.0004	72,735.73	_				
	Olive	7.68	0.22	0.024	0.0003	0.0014	16,736.00	0.0020	0.08	0.009	0.0005	544,233.72	1,668,369.75	0.06	62.5	6.79	0.0008	30,928.12					
Total *		10.78	0.22	0.03	0.0004	0.0019	12,444.78	0.0027	0.08	0.012	0.0006	404,688.61	1,240,588.75	0.08	62.5	9.13	0.0011	22,997.95					
Legumes and Pulses	Peas	1.02	_						0.01	0.0001	0.00001	31,975,034.40	98,020,718.55	0.001	_				_				
	Beans	12.82							0.01	0.002	0.0001	2,590,207.23	7,940,381.57	0.01									
	Lentils	25.52							0.05	0.02	0.001	258,357.36	792,004.59	0.13									
	Chickpeas	19.05							0.015	0.004	0.0002	1,210,257.84	3,710,092.75	0.03									
	Green fava beans and green peas	8.37							0.01	0.001	0.0001	4,024,072.28	12,335,950.97	0.01									
Total *		66.78							0.019	0.017	0.001	275,071.74	843,243.17	0.12									
Milk and dairy products	Milk	50.64	_						_						_				0.0225	0.02	0.0828	34,423.06	0.0001
	Yogurt	71.95																	0.0572	0.06	0.2912	9788.10	0.0005
	Labneh	24.59																	0.2	0.07	0.3496	8153.18	0.0006
	Cheese	5.08																	0.0471	0.003	0.0174	164,054.09	0.00003
	Kareche	0.68																	0.8282	0.01	0.0377	75,498.26	0.0001
	Milk based ice cream	7.60																	0.025	0.003	0.0134	212,888.18	0.00002
	Milk based pudding	7.49																	0.025	0.003	0.0136	209,937.47	0.00002
Total *		168.03																	0.17	0.39	1.96	1454.06	0.003

* Total was calculated as the sum of mean dietary intake of every food item.

**Table 4:** Dietary intake, EDI of analyzed mycotoxins and risk assessment components (MOE, HQ, liver cancer cases, weekly exposure) in minor food groups including herbs, spices and condiments; snacks and desserts; traditional food; beverages; and nuts and oilseeds.

			Mycotoxins															
			AFB1						OTA						DON			
Food Groups	Food Items	Mean Dietary Intake (g/day)	Mean AFB1 (µg/kg)	EDI (ng/kg bw/day)	HQ Lower Limit	HQ Upper Limit	MOE	Liver Cancer Risk (Cancer Cases/100,000 Persons)	Mean OTA (µg/kg)	EDI (ng/kg bw/day)	HQ	MOE non-neo	MOE neo	Weekly Exposure (ng/kg bw)	Mean DON (µg/kg)	EDI (ng/kg bw/day)	HQ	MOE
Herbs, spices and condiments	Thyme	20.54	0.52	0.15	0.0019	0.0090	2624.32	0.0127	0	0	0	0	0	0	_			
	Seeds	1.07	0.22	0.003	0.00004	0.0002	118,651.72	0.0003	0.08	0.001	0.0001	3,858,405.51	11,828,093.01	0.009	62.5	0.96	0.0001	219,268.37
Total *		21.61	0.37	0.108	0.00132	0.0064	3690.71	0.0090	0.04	0.012	0.0007	403,694.19	1,237,540.32	0.082	62.5	18.31	0.0023	11,470.72
Snacks and desserts	Pastries	1.01	0.11	0.002	0.00002	0.0001	259,834.93	0.0001	0.15	0.002	0.0001	2,253,201.86	6,907,278.44	0.01	109.67	1.53	0.0002	136,823.81
	Biscuit	1.92	_						0.71	0.02	0.0011	236,026.57	723,548.67	0.14	31	0.87	0.0001	240,002.46
	Cakes	3.17							0.455	0.02	0.0012	226,544.14	694,479.91	0.15	60	2.75	0.0003	76,273.05
	Croissant	1.44							0.505	0.01	0.0006	443,316.63	1359004.46	0.07	50	1.06	0.0001	198,789.55
	Doughnut	0.90							0.34	0.004	0.0002	1,109,254.75	3,400,463.83	0.03	60	0.75	0.0001	279,072.55
	Chocolate	3.72							0.025	0.001	0.0001	3,436,667.33	10,535,238.12	0.010	_			
Total *		12.17	0.11	0.018	0.0002	0.0011	22,054.64	0.0015	0.364	0.06	0.0033	78,775.96	241,490.79	0.42	62.134	10.24	0.0013	20,498.53
Traditional food	Kishik	10.53	0.83	0.12	0.0015	0.007	3297.74	0.01	1.14	0.167	0.0093	28,391.65	87,035.71	1.17	_			
	Kibbeh	9.12	_						0.025	0.003	0.0002	1,443,453.85	4,424,964.22	0.02				
	Meat pie	2.23							0.51	0.016	0.0009	295,927.95	907,178.71	0.11	88	2.76	0.0003	76,143.29
Total *		21.88	0.83	0.25	0.0030	0.014	1625.16	0.02	0.56	0.166	0.0092	28,568.22	87,576.98	1.16	88	26.10	0.0033	8047.34
Alcoholic beverages	Spirits and alcohol	0.60	_						1.47	0.01	0.0006	468,475.62	1,436,130.33	0.07	52.08	0.36	0.00004	587,072.19
Stimulants	Coffee	73.23							0.51	0.53	0.0296	8867.94	27,185.01	3.73	_			
Nuts and oilseeds	Nuts	0.46	0.4	0.0025	0.00003	0.0001	157,224.95	0.0385	0.25	0.002	0.0001	2,974,696.01	9,119,046.98	0.01				

* Total was calculated as the sum of mean dietary intake of every food item.

**Table 5:** Co-occurrence of mycotoxins in the Lebanese food basket.

	Mycotoxins															
	AFB1				AFM1				OTA					DON		
Food Groups	Total EDI (ng/kg bw/day)	Total HQ	MOE	Total Liver Cancer Risk (Cancer Cases/100,000 Persons)	Total EDI (ng/kg bw/day)	Total HQ	Total MOE	Total Liver Cancer Risk (Cancer Cases/100,000 Persons)	Total EDI (ng/kg bw/day)	Total HQ	Total MOE non-neo	Total MOE neo	Total Weekly Exposure (ng/kg bw)	Total EDI (ng/kg bw/day)	Total HQ	Total MOE
Cereals and cereal based products	0.86	0.0105 ^a^–0.0504 ^b^	466.59	0.0712	NA	NA	NA	NA	3.29	0.183	1439.47	4412.76	23.00	347.05	0.0434	605.11
Snacks and desserts	0.018	0.0002 ^a^–0.0011 ^b^	22,054.64	0.0015	NA	NA	NA	NA	0.06	0.0033	78,775.96	241,490.79	0.42	10.24	0.0013	20,498.53
Herbs, spices and condiments	0.108	0.00132 ^a^–0.0064 ^b^	3690.71	0.0090	NA	NA	NA	NA	0.012	0.0007	403,694.19	1,237,540.32	0.082	18.31	0.0023	11,470.72
Fruits and Fruit Products	0.03	0.0004 ^a^–0.0019 ^b^	12,444.78	0.0027	NA	NA	NA	NA	0.012	0.0006	404,688.61	1,240,588.75	0.08	9.13	0.0011	22,997.95
Milk and Dairy products	NA	NA	NA	NA	0.39	1.96	1454.06	0.003	NA	NA	NA	NA	NA	NA	NA	NA
Nuts and Oilseeds	0.0025	0.00003 ^a^–0.0001 ^b^	157,224.95	0.0385	NA	NA	NA	NA	0.002	0.0001	2,974,696.01	9,119,046.98	0.01	NA	NA	NA
Legumes and Pulses	NA	NA	NA	NA	NA	NA	NA	NA	0.017	0.001	275,071.74	843,243.17	0.12	NA	NA	NA
Traditional food	0.25	0.0030 ^a^–0.014 ^b^	1625.16	0.02	NA	NA	NA	NA	0.166	0.0092	28,568.22	87,576.98	1.16	26.10	0.0033	8047.34
Alcoholic Beverages	NA	NA	NA	NA	NA	NA	NA	NA	0.01	0.0006	468,475.62	1,436,130.33	0.07	0.36	0.00004	587,072.19
Stimulant Beverages	NA	NA	NA	NA	NA	NA	NA	NA	0.53	0.0296	8867.94	27,185.01	3.73	NA	NA	NA
**Total**	1.26	0.0154 ^a^–0.0744 ^b^	316.30	0.105	0.39	1.96	1454.06	0.003	4.10	0.228	1154.45	3539.00	28.68	411.18	0.0514	510.72

NA: Data not available in the study. ^a^: Lower limit of HQ. ^b^: Upper limit of HQ.

**Table 6:** Total estimated dietary exposure to AFB1, AFM1, OTA and DON for different age groups in Lebanon and risk assessment components.

	AFB1				AFM1				OTA					DON		
Age Categories	Total EDI (ng/kg bw/day)	Total HQ	Total MOE	Total Liver Cancer Risk (Cancer Cases/100,000 Persons)	Total EDI (ng/kg bw/day)	Total HQ	Total MOE	Total Liver Cancer Risk (Cancer Cases/100,000 Persons)	Total EDI (ng/kg bw/day)	Total HQ	Total MOE non-neo	Total MOE neo	Total Weekly Exposure (ng/kg bw)	Total EDI (ng/kg bw/day)	Total HQ	Total MOE
Older Adolescents (18–19 years)	1.05	0.01 ^a^–0.06 ^b^	382.30	0.09	0.16	0.82	3467.83	0.001	4.05	0.22	1168.28	3581.39	28.34	493.73	0.06	425.33
Young Adults (20–24 years)	1.06	0.01 ^a^–0.06 ^b^	377.51	0.09	0.16	0.81	3536.26	0.001	4.15	0.23	1140.28	3495.58	29.04	480.33	0.06	437.20
Adults (25–59 years)	0.89	0.01 ^a^–0.05 ^b^	451.20	0.07	0.16	0.80	3557.66	0.001	3.40	0.19	1389.75	4260.33	23.82	393.70	0.05	533.40
Older Adults (≥60 years)	0.79	0.01 ^a^–0.05 ^b^	505.54	0.07	0.17	0.84	3386.87	0.001	3.23	0.18	1464.11	4488.28	22.61	356.59	0.04	588.91
Total Adults (18 till ≥ 60 years)	0.95	0.011 ^a^–0.055 ^b^	422.87	0.079	0.16	0.82	3485.87	0.001	3.71	0.206	1275.71	3910.74	25.95	431.09	0.054	487.14

^a^: Lower limit of HQ. ^b^: Upper limit of HQ.

**Table 7:** Food items included in the study and mycotoxins analyzed in each food item.

	Mycotoxins Analyzed					
Food Items	AFB1	AFM1	OTA	OTB	DON	T2/HT-2
Arabic bread	✓	✗	✓	✓	✓	✓
French Baguette, Cooked bulgur, Keshek, Thyme, Nuts	✓	✗	✓	✗	✗	✗
Toast, Lebanese Kaak, Cornflakes, Pasta, Dried fruits, olives, seeds, Pastry, Pie	✓	✗	✓	✗	✓	✗
Cooked Rice, Kibbeh, Pulses and Legumes (Peas, Beans, Chickpeas, lentils), Chocolate, Caffeinated beverages	✗	✗	✓	✗	✗	✗
Milk and Dairy products (Yogurt, Labneh, cheese, karishe, milk based ice cream and pudding)	✗	✓	✗	✗	✗	✗
Biscuit, Cake, Pizza, Manaeech, Meat pie, Croissant, Donut, Alcoholic beverages	✗	✗	✓	✗	✓	✗
Oil	✓	✗	✗	✗	✗	✗

✓ Analyzed, ✗ Not analyzed.

**Table 8:** Ranges of LOD, LOQ and recovery rates for each mycotoxin and food group.

		LOD (μg/kg)	LOQ (μg/kg)	Recovery Rate
AFB1	Arabic bread	0.0042	0.027	88%
	French Baguette, Cooked bulgur, Keshek, Thyme, Nuts	0.0042	0.027	88%
	Toast, Lebanese Kaak, Cornflakes, Pasta, Dried fruits, olives, seeds, Pastry, Pie	0.0042–0.01	0.027–0.03	88–96%
	Oil	0.01	0.03	96%
AFM1	Milk and Dairy products (Yogurt, Labneh, cheese, karishe, milk based ice cream and pudding)	0.001–0.005	0.01–0.03	74–98%
OTA	Arabic bread	0.00012–1	0.00035–0.015	94–97%
	French Baguette, Cooked bulgur, Keshek, Thyme, Nuts	0.0034–0.5	0.015	94%
	Toast, Lebanese Kaak, Cornflakes, Pasta, Dried fruits, olives, seeds, Pastry, Pie	0.0034–1	0.015–0.21	75–94%
	Cooked Rice, Kibbeh, Pulses and Legumes (Peas, Beans, Chickpeas, lentils), Caffeinated beverages	0.05	0.21–0.8	75–86%
	Biscuit, Cake, Pizza, Manaeech, Meat pie, Croissant, Donut, Alcoholic beverages, Chocolate	0.05–1	0.21	75%
OTB	Arabic bread	0.06	0.18	93%
DON	Arabic bread	30		
	Toast, Lebanese Kaak, Cornflakes, Pasta, Dried fruits, olives, seeds, Pastry, Pie	30–62.5	125	85%
	Biscuit, Cake, Pizza, Manaeech, Meat pie, Croissant, Donut, Alcoholic beverages, Chocolate	30–62.5	125	85%
T2	Arabic bread	0.39	1.19	105%
HT2	Arabic bread	0.83	2.52	100%

## Data Availability

The data presented in this study are available within this article.
